# COVID-19-Induced Eosinophilic Lower Airway Inflammation in Those With Multiple COVID-19 Vaccinations

**DOI:** 10.7759/cureus.38368

**Published:** 2023-05-01

**Authors:** Yosuke Fukuda, Hidenori Hayashi, Hironori Sagara

**Affiliations:** 1 Department of Medicine, Division of Respiratory Medicine and Allergology, Yamanashi Red Cross Hospital, Yamanashi, JPN; 2 Department of Medicine, Division of Respiratory Medicine and Allergology, Showa University School of Medicine, Tokyo, JPN; 3 Department of Medicine, Division of Respiratory Medicine and Allergology, Showa University Koto Toyosu Hospital, Tokyo, JPN

**Keywords:** viral infection, vaccine, eosinophilic pneumonia, eosinophilia, covid-19

## Abstract

A 29-year-old woman was admitted with a diagnosis of ischemic enteritis. She had a coronavirus disease 2019 (COVID-19) infection four weeks before this visit and continued to experience a cough. Four months before, she received the third COVID-19 vaccine. Chest computer tomography revealed scattered ground-glass opacities in both upper lobes. Based on abnormalities in chest imaging, eosinophilia, and a high level of fractional exhaled nitric oxide, she was diagnosed with eosinophilic lower airway inflammation due to COVID-19. Since the visit, the patient had an intermittent fever and no radiological improvement, so systemic corticosteroid treatment was initiated, and the symptoms and clinical findings improved. Clinicians should know the potential association between COVID-19 and eosinophilic lower airway inflammation, which may still occur despite multiple vaccinations.

## Introduction

Currently, although approximately 70% of the world's population has received at least one dose of a novel coronavirus disease 2019 (COVID-19) vaccine [1], the number of people affected by the virus remains high. Despite the benefits of vaccination and the mutation of severe acute respiratory syndrome coronavirus 2 (SARS-CoV-2), which have reduced the likelihood of COVID-19 becoming a severe disease, allergic reactions due to infection or vaccination may still occur and can be severe. In vitro, viral infection-induced eosinophilic airway inflammation was proved [2]. Herein, we present a case of eosinophilic lower airway inflammation triggered by COVID-19 after the patient received multiple vaccinations against the virus.

## Case presentation

**Figure 1:**
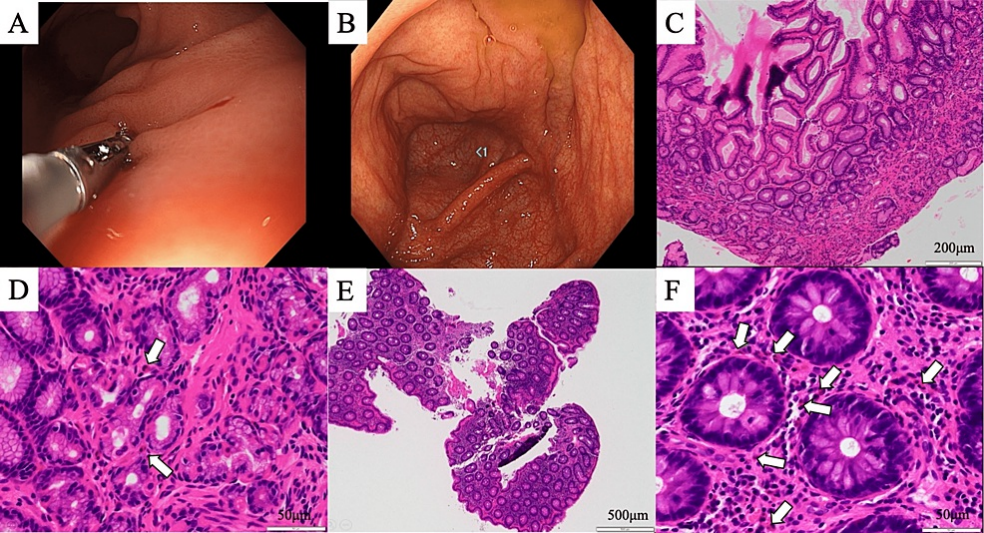
Endoscopic and pathologic findings of the upper and lower gastrointestinal tracts Endoscopic findings on esophagogastroduodenoscopy (A) and colonoscopy (B) showed no mucosal redness or ulcerative lesions. Histopathology findings on esophagogastroduodenoscopy and colonoscopy showed no evidence of granulomatous or fibrinoid necrotizing vasculitis of the microvasculature but mild eosinophilic infiltration of the surrounding tissue (white arrows). Stomach (hematoxylin and eosin staining, 10× magnification) (C), stomach (hematoxylin and eosin staining, 40× magnification) (D), ascending colon (hematoxylin and eosin staining, 10× magnification) (E), and ascending colon (hematoxylin and eosin staining, 40× magnification) (F).

A 29-year-old, never-smoker female patient presented to the emergency department with a chief complaint of abdominal pain and bloody stools that gradually worsened over a half-day period. The patient's past medical history was significant for cedar and cypress pollinosis, as well as a four-week history of COVID-19 infection prior to this visit complicated by a persistent cough. The patient denied any drug or food allergies or recent changes in medications. Four months prior to the presentation, she had received the third Pfizer-BioNTech COVID-19 vaccine. The patient's vital signs during the visit were as follows: clear consciousness, body temperature 36.0°C, pulse rate 87 beats per minute, blood pressure 136/86 mmHg, respiratory rate 16 breaths per minute, and oxygen saturation 97% in ambient air. Relevant physical examination findings included tenderness in the mediolateral portion of the left lower quadrant of the patient's abdomen, normal respiratory sounds, and absence of skin rashes on the extremities and trunk. Abdominal computed tomography (CT) revealed thickening of the intestinal wall from the ileum to the sigmoid colon. A clinical diagnosis of ischemic enteritis was made, and the patient was admitted to the emergency department. The patient underwent esophagogastroduodenoscopy and colonoscopy. Endoscopic findings revealed no disease-specific findings (Figures 1A, 1B).

**Figure 2:**
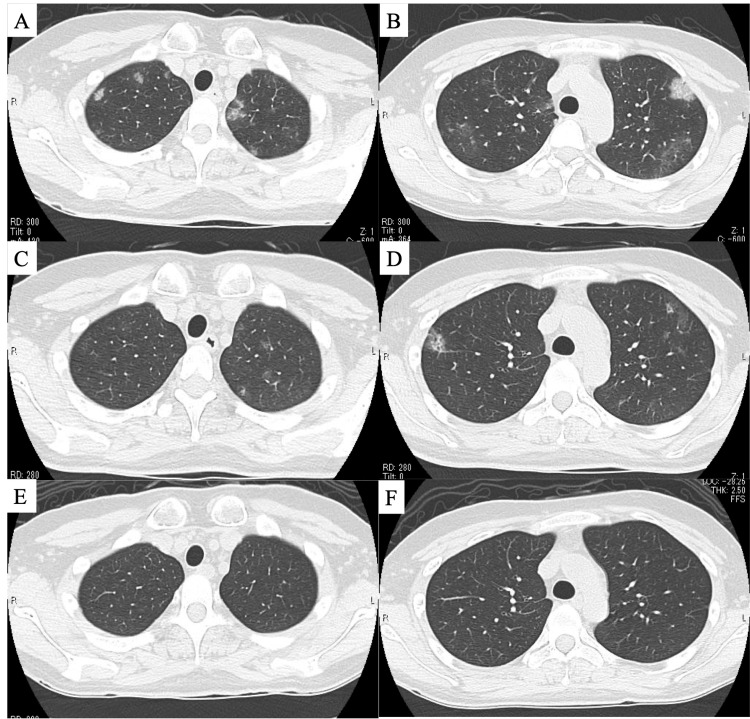
Radiological findings before and after treatment Chest computed tomography showed scattered frosted ground-glass opacities predominantly inferior to the pleura in the bilateral upper lobes on admission (A, B). These findings remained three weeks after initial presentation (C, D), and improved with the administration of systemic corticosteroids (E, F).

In addition, histopathology showed no evidence of granulomatous or fibrinoid necrotizing vasculitis of the microvasculature but mild eosinophilic infiltration of the surrounding tissue (Figures 1C-1F). In addition, chest CT revealed scattered ground-glass opacities, predominantly inferior to the pleura in both upper lobes (Figures 2A-2B).

The patient's peripheral blood eosinophil count increased during admission, with the highest value reaching 1380 cells/μL (white blood cell 9670 cells/μL). The serum antineutrophil cytoplasmic antibody test was negative. Although the patient's abdominal symptoms improved, she continued to experience a cough and newly intermittent fevers. The fractional exhaled nitric oxide (FeNO) concentration was 74 ppb, and the respiratory function test revealed forced vital capacity (FVC) was 3.43L (102.7% of the predicted value), and forced expiratory volume in 1 second (FEV1) was 2.91L (99.3% of the predicted value). Inhaled corticosteroids (once-daily fluticasone furoate/vilanterol 100 μg/25 μg) were started to manage the patient's eosinophilic airway inflammation, but intermittent fever and abnormal radiological findings remained (Figures 2C-2D). One month later after the visit, a bronchoscopy was performed at the right upper lobe, and bronchoalveolar lavage (BAL) fluid revealed lymphocytosis (56%) and no eosinophils (0%) with a CD4+/CD8+ ratio of 2.8. The tissue diagnosis was challenging due to coughing during the examination. Based on the clinical course, the patient was diagnosed with eosinophilic lower airway inflammation related to COVID-19, and systemic corticosteroid treatment was initiated. Treatment was started at an initial dose of 30 mg prednisolone equivalent and tapered off at a rate of 5 mg/two weeks for three months. After treatment, the patient's cough, fever, and chest CT findings improved (Figures 2E-2F). Subsequently, respiratory function tests were re-performed when the patient was free of inhaled and systemic steroids revealed that the FVC) was 3.47L (102.7% of the predicted value), and FEV1 was 2.93L (100.0% of the predicted value). The FEV1/FVC% was 84.4%, and the V50/V25 was 2.16. In addition, there was no significant airway reversibility, with only a slight improvement in FEV1 of 230 ml (7.9%) after the inhalation of a short-acting β2-agonist. The patient was monitored after treatment, and no clinical symptoms recurred.

## Discussion

Previous reports have described cases of eosinophilic lower airway inflammation, represented by eosinophilic pneumonia following COVID-19 infection, and none of them had received the COVID-19 vaccine or had no information about it (Table 1) [3-6].

**Table 1:** Clinical characteristics of eosinophilic lower airway inflammation caused by COVID-19 BALF, bronchoalveolar lavage fluid

Case	Age	Sex	Country	Year	Eosinophils in blood(%)	Eosinophils in BALF (%)	Vaccination	References
1	29	F	Japan	2022	1383	0	mRNA (three doses)	This case
2	60	M	USA	2020	0	37	−	3
3	61	M	Spain	2021	200	5	−	4
4	77	M	Japan	2021	1016	−	−	5
5	51	M	Portugal	2022	8840	94	−	6

To our knowledge, this is the first reported case of eosinophilic lower airway inflammation triggered by COVID-19 infection despite multiple vaccinations. SARS-CoV-2 infection promotes a T-helper 2 inflammatory response, leading to a persistent elevation of eosinophilic inflammation-associated factors, such as CCL11 and IL-5, even two weeks after COVID-19 infection [7]. Therefore, eosinophilic lower airway inflammation should be considered as a potential complication of COVID-19 infection, particularly in patients with a predisposition to allergy, as in this case.

There are several limitations to this report. First, the eosinophil count in the BAL fluid was not elevated, which may have been due to the use of inhaled steroids before bronchoscopy. In addition, the fact that there were four weeks between the initial presentation and the bronchoscopy could have had an impact. Although eosinophil infiltration of lung tissue could not be confirmed and organic pneumonia after COVID-19 could not necessarily be ruled out as a differential diagnosis, a diagnosis of eosinophilic lower airway inflammation was considered reasonable based on abnormalities in chest imaging and elevated FeNO level before treatment [8]. Second, the patient's blood eosinophil count did not increase significantly. It was presumed that desensitization may have been established and allergic reactions suppressed by multiple vaccinations, but further verification is needed. The patient had never previously been noted to have eosinophilia in the blood, and follow-up blood tests again showed no eosinophilia in the blood. Thus, we were reasonable in determining that there was an association between blood eosinophilia and COVID-19. Third, the possibility of eosinophilic granulomatosis with polyangiitis (EGPA) cannot be ruled out because the patient presented with abdominal pain and bloody stools at the initial examination. Although tissue biopsies of the upper and lower gastrointestinal tracts did not show prominent eosinophilic infiltrates or granulomas, pathological findings, including the gastrointestinal tract, alone cannot be used to exclude EGPA as a diagnosis [9]. In previous reports, there were several cases of eosinophil infiltration of the upper and lower gastrointestinal tracts triggered by COVID-19 infection or vaccination, but the detailed mechanism is unclear [10,11]. Furthermore, since no challenge test has been performed, the presence of latent asthma cannot be ruled out, which is another reason why EGPA remains a differential diagnosis. Several cases of EGPA triggered by COVID-19 were also reported [12-14]. Therefore careful monitoring of disease progression, including the presence of new neurological symptoms, is necessary.

## Conclusions

In conclusion, clinicians should be aware of the potential association between COVID-19 and eosinophilic lower airway inflammation, which may still occur despite multiple vaccinations, especially with a predisposition to allergy.
